# PD06 - Early elevated blood eosinophils are predictive for the development of atopic dermatitis in an atopic birth cohort

**DOI:** 10.1186/2045-7022-4-S1-P6

**Published:** 2014-02-28

**Authors:** Siri Rossberg, Kerstin Gerhold, Georg Menke, Kurt Zimmermann, Thomas Geske, Stock Philippe, Susanne Lau

**Affiliations:** 1 Department for Pediatric Pneumology and Immunology, Charité University Hospital, Berlin, Germany; 2 GE-ME Design and Analyse Klinischer Prüfung, Frankfurt, Germany; 3 SymbioPharm GmbH, Herborn, Germany; 4 TG Medical Services, Berlin, Germany

## 

The value of predictive markers for the development of atopic disease has long been discussed. Contributing to the discourse, we investigated the role of blood eosinophils at 4 and 30 weeks of life and their association with developing atopic dermatitis (AD) in an atopic birth cohort of 606 children enrolled in a randomized placebo controlled trial for prevention of AD.

## Methods

The infants received the placebo controlled oral treatment with a three times daily applied bacterial lysate (ProSymbioflor^®^: Escherichia coli and Enterococcus faecalis) from week 5 until 7 months of life and were followed-up until 3 years of life. Blood samples for eosinophil counts were taken at 4-5 weeks and 7 months of life. Elevation of blood eosinophils was defined as counts above 5% of total leukocytes.

## Results

At 4 weeks of life and 7 months of life, respectively, 233/559 and 107/467 infants showed elevated blood eosinophils counts in the total study group. Elevated blood eosinophils observed at 4 weeks were significantly associated with the occurrence of AD in the whole study group at the time points 7 months (p =0.0073), one year (p=0.0035), two years (p=0.0069) and three years (p=0.006) of life. This observation was seen in the active group as well as the placebo treated group. Blood eosinophil counts at 7 months of life showed only borderline significance for developing AD (p=0.06) at the same age, and blood eosinophil counts at one year of life showed no association with AD.

## Conclusion

Elevated blood eosinophils at age 4 weeks of life seem to be of predictive value for the onset of atopic dermatitis in infancy and early childhood in a high risk birth cohort. Eosinophil counts later in infancy were less correlated with AD prevalence. Early eosinophil counts can therefore be helpful for counseling the parents but furthermore can identify target groups for interventional trials aiming at allergy prevention.

